# Medication management in the context of mental illness: an exploratory study of young people living in Australia

**DOI:** 10.1186/s12889-020-09237-9

**Published:** 2020-07-30

**Authors:** Sara S. McMillan, Victoria Stewart, Amanda J. Wheeler, Fiona Kelly, Helen Stapleton

**Affiliations:** 1 School of Pharmacy and Pharmacology, Quality Use of Medicines Network, Menzies Health Institute Queensland, Griffith University, Gold Coast, Australia; 2 School of Human Services and Social Work, Menzies Health Institute Queensland, Griffith University, Brisbane, Australia; 3 Faculty of Medical and Health Sciences, University of Auckland, Auckland, New Zealand

**Keywords:** Young people, Medication experience, Mental illness, Qualitative, Pharmacy

## Abstract

**Background:**

Young people face significant challenges when managing a mental illness, such as acquiring treatment autonomy, being inexperienced users of the healthcare system and associated peer-related stigma. While medication use can be challenging in its own right, there is comparatively little information about the associated experiences and needs of young people with mental illness, particularly in the Australian context. This exploratory study will provide valuable insight into how this group is currently supported in relation to medication use.

**Methods:**

Young people (aged 14–25 years) who had used a prescription medication for any mental illness for a minimum of 2 months were eligible to participate in this qualitative exploratory study. Semi-structured interviews were conducted between October 2017–September 2018 in consultation rooms at two youth-focused mental health support organisations in Brisbane, Queensland. Interview questions explored how participants managed their medication and related experiences. Interviews were transcribed verbatim and descriptively analysed using thematic analysis.

**Results:**

Eighteen young people discussed their lived experience during interviews averaging 50 min in duration. Finding the right medication that reduced symptom severity with minimal side-effects was identified as a complex experience for many, particularly when there was a lack of information, support or reduced financial capacity. Young people described a range of strategies to manage medication side-effects, changes and to support routine medication use.

**Conclusions:**

Young people persevered with taking medication to manage a mental illness within a healthcare system that does not adequately support this vulnerable population. There remains a clear directive for healthcare professionals to provide credible information that proactively engages young people as healthcare participants, and for policy makers to consider financial burden for this population with limited financial capacity.

## Background

Mental illness is suggested to affect up to one in five children and adolescents worldwide [1] and is of significant public health concern. Determining rates of mental illness amongst young people remains somewhat elusive, given the heterogenous nature of the multitude of studies reporting prevalence data [2]. Regardless, supporting the mental health of young people, in addition to their physical wellbeing, remains a key priority in many countries, including Australia. It is estimated that 14% of Australian school students (aged 4–17 years) have a mental illness [3]. This prevalence has prompted the Australian Government to provide public health policy initiatives and youth-specific mental health services [4]. However, further attention is needed. A 2018 survey of 28,000 young Australians (15–19 years) identified that mental health was the most important issue of personal concern, and mental wellbeing was closely related to coping with stress, managing study problems and maintaining a positive body image [5]. While mental illness is not the sole instigator of suicidality [6], suicide is currently reported as the leading cause of death in young Australians between 15 and 24 years [7].

Young people with a mental illness are a vulnerable group in different ways to their older counterparts. Age and developmental differences mean that they have different health and communication needs to those of older people [8]. Young people make health decisions differently to older people, and experience barriers to healthcare that can include inexperience and lack of knowledge about access, out-of-pocket costs, stigma and negative peer attitudes [9]. Research exploring decision-making surrounding service use by young people experiencing mental illness has been previously conducted [10]. This is important given the recognised concerns around youth engagement with mental health services.

Adolescence is also a time of shifting from a focus on family to greater independence and identification with peers [11]. This can require the young person to balance autonomy in treatment decisions with the opinions and wishes of parents. Overall, young people experience a range of challenges when seeking and receiving care for mental illness [12]. Embarrassment about seeking help and poor self-worth due to the internalization of stimatizing messages about mental illness are reported [12, 13]. The impact of discrimination and stigmatization of young people with mental illness has been found to be significant and universal, impacting on help-seeking behaviour [14]. In addition, qualitative studies have highlighted young people’s concerns regarding medication dependency [15] and the importance of a collaborative approach with a trusted clinician when deciding to commence or change medication [16].

Increases in psychotropic medication use, such as antidepressants and antipsychotics, have been noted in young people over the last decade [17, 18]. Over 2.3 million mental health prescriptions (subsidised and under the co-payment of the Pharmaceutical Benefits Scheme, PBS^1^) were dispensed between 2017 and 18 for Australian youth aged 15–24 years [19]. The experience of using psychotropic medication can be both positive and negative, as evidenced by a recent narrative review of the qualitative literature [20]. Whilst medications are identified as helpful in the management of symptoms [16], their use may inadvertently advertise a young person’s mental illness to others, setting them apart from their peers [21]. Treatment burden, or the workload and lifestyle impact imposed upon the person managing their health condition, has been identified as an important factor influencing medication use [22]. For example, the experiences of using medication and the associated burden may influence medication adherence, and hence, health and wellbeing outcomes [23]. While there is increasing literature on the impact of treatment burden, this has been predominantly focused on older people living with a range of chronic illnesses [23, 24]. There is comparatively little information on the needs of young people managing their psychotropic medication, particularly within the Australian context. Kranke and colleagues have published some important work in the United States context, albeit from the same data-set [21, 25–27]. There were benefits and risks associated with psychotropic medication use, such as improved grades and “zombie feelings” [27], and specific concerns relating to stigma [21, 25, 26]. Furthermore, many studies using qualitative approaches have focused on one specific mental illness, such as depression [28], or medication group, for example, antipsychotics [29, 30] or antidepressants [31].

Important questions remain about how young people manage and experience psychotropic medication use. This study aimed to explore the experiences of young Australians taking and managing medication for any mental illness. It formed part of a larger project that specifically examined the need for, and features of, a youth-friendly pharmacy service for this vulnerable population, thereby providing further guidance on policy and service delivery.

## Methods

A qualitative approach, using semi-structured interviews and descriptive thematic analysis, was chosen as the most appropriate means for participants to provide narrative responses, i.e. to share their medication experiences in their own words [32].

### Participants and recruitment

Young people (aged 14–25) were purposively recruited from two non-government services in Brisbane, Queensland that provide youth-oriented mental health support. Support includes access to healthcare professionals, such as general practitioners (GPs) and psychologists, and assistance in relation to mental and physical health, alcohol and other drug use, and study/employability services. The Youth Reference Group from one service assisted with co-designing study promotional material and provided feedback on the information sheet and recruitment processes. Clinical intake teams from both services screened their clientele for potential participants, provided study information, and obtained expressions of interest. Young people could also independently contact the research team, who assessed eligibility and provided further information as needed.

Inclusion criteria required that the young person had a mental illness for which they had been taking a prescribed medication for a minimum of 2 months, were living in the community with regular contact with a healthcare professional and were able to provide written informed consent. All participants under 18 years required additional parental/guardian consent. A follow-up phone call from the researchers to participants answered any further questions and confirmed an interview time at the service attended by the young person.

A range of strategies were used for participant recruitment, including social media, therefore the response rate was unable to be determined. A total of 24 young people expressed interest in the study; six young people were not interviewed for a variety of reasons, i.e. they were no longer interested, an interview time was unable to be scheduled, or they did not respond to a follow-up phone call.

### Data collection

A semi-structured interview guide (Table 1) was informed by the literature [33–37] and feedback from two service clinicians and the Youth Reference Group. Interview questions were piloted with three researchers (VS, FK, AW) and a consumer with lived experience of mental illness and medication use. Questions were non-directive, with prompts to introduce research-related domains if these did not arise spontaneously. The guide was emailed to all participants prior to the interview to ensure that they were comfortable with the nature of the questions being asked. Participants were reminded of the reasons for the study at interview commencement. Participants were advised that they did not need to disclose specific details of their medication or mental illness/es but rather, were given the opportunity during the conversation to volunteer this information during the interview. While noted as a limitation, this approach was taken to mitigate potential distress arising from talking to a stranger.

**Table 1:** Interview Guide

*Main focus*	*Interviewer questions*
Introduction and rapport development	Introductions, thanks for participating, ask if comfortable with venue, describe purpose of interview.
Ensure participant well enough for participation	Explain process of screening questions and undertake health screening questionnaire, ensure results demonstrate eligibility.
Informed consent	Review information sheet, answer questions and confirm participant understanding. Complete consent forms and obtain parental/guardian consent if required.
Find out experience of taking medication to manage mental illness	What do you think about taking medication for mental illness? What are the benefits from taking medication for mental illness? How involved were you in the decision to start medication? How did you feel when you first started to take medication for your mental illness?
Find out how young people manage their medication	Can you describe how you manage your medication? Do you ever change how you take your medication? (if yes, in what situations?) Do you have any concerns with taking your medication? If managing own medication: When did responsibility of medication transfer to you or did you always have control? Why do you think this happened? When would responsibility of managing your medication change? Where would you go / who would you talk to if you wanted advice on your medication?
Completing the interview	Summarise discussion to confirm understanding, reminder of contacts on information sheet including support options, ask support person in the room if they would like to add anything, determine if the young person would like a copy of the transcript, provide gift voucher / thanks.

All interviews took place in private consultation rooms at each service. Participants were able to bring a support person or family member, with two (both under the age of 16 years) choosing this option. A strategy was in place to screen for, and manage, any emotional distress arising from the use of a brief mental health assessment, which participants were advised of prior to the interview [38]. Written consent was obtained immediately prior to starting interviews, including consent to audio-record the interview. Interviews occurred between October 2017 and September 2018 and averaged 49.65 min (range: 21.41–70.34). A $25 movie voucher was provided to participants alongside an opportunity to debrief with a clinician at the end of the interview, as required. Ethics approval was obtained from a University Human Research Ethics Committee (Ref No: 2017/348).

### Data analysis

All interviews were audio-recorded and transcribed verbatim. All transcripts were read by the first and last authors (SM, HS); a pharmacist and midwife/social scientist respectively. The first author subsequently engaged in multiple readings of each transcript to familiarise herself with the overall, and individual, content, identify primary themes across the complete dataset, and establish a preliminary coding frame. This coding frame was informed by a review of the literature [20] and the Medication Experience Model [39]. The Medication Experience Model interrelates the concepts of illness experience, medication acceptance and medication experience; specifically benefits, side effects, burden, adherence and alternative or additional support [39]. The authors were familiar with the model as they had previously used it to guide the coding of a narrative literature review on a similar topic [20]. However, the analysis did not solely rely on a deductive approach, with themes allowed to emerge from the data (i.e. inductive coding).

As the study was focused on other aspects of medication management, such as experiences obtaining medication from a community pharmacy, other data emerged that is reported elsewhere [40]. Following this period of concentrated data immersion, results were compared, anomalies resolved, and the dominant categories agreed upon by both researchers (SM, HS). Similar ideas were categorised into a single code, for example, all information related to medication management; these codes were then further refined into sub-themes, such as adherence, other substance use and treatment burden (i.e. axial coding). Key themes were then derived and named, using “content-characteristic words” [32]. Data were managed using the software package NVivo© (version 12, QSR International Pty Ltd., Doncaster, Victoria, Australia) which enabled sophisticated filing, easy retrieval and complex interrogation across the dataset. Some of the minor themes and diverse cases identified in the analysis have been presented in the results; other themes are reported in another paper focused on pharmacy experiences [40].

Key procedures undertaken by researchers ensured study rigor and trustworthiness. To ensure consistency in study processes the first author, an experienced qualitative researcher in pharmacy practice, conducted most interviews. This provided extensive opportunities for revisiting the shared interview experience and allowed for an initial analysis of relevant contextual data, including respondent voice tone, and hesitancy/confidence in answering questions. Debriefing and updating the research team was achieved through the sharing of written summaries which the first author wrote immediately following the conclusion of each interview. The summaries provided an overview of key findings from each interview and any important contextual information to facilitate further discussions with the research team.

The initial coding framework was further developed with input from the last author, whose different clinical and academic background provided corresponding, and alternative, perspectives [41]. The interviewer was a registered pharmacist, however, did not disclose this information to participants unless asked. A sample of transcripts were quality checked by two other researchers (VS, FK); this involved listening to the audio-recording and confirming that the transcript was a verbatim account of the interview. Participants were offered a copy of their individual transcript. Eight participants initially requested this in-person, however only two participants responded to a follow-up email from the research team; no changes were made to the transcripts. It was proposed that the findings would be presented to the Youth Reference Group to obtain their interpretation and invite their feedback. However, due to the frequently changing membership of this body, there was limited opportunity to establish a working relationship with members and to follow through with this plan. Time constraints, no a priori relationship with participants, and the one-off nature of interviews also worked against inviting participants to provide feedback. Findings were not checked by participants. In the following results section, key themes are illustrated by quotations from individual participants (P1, P2, etc.) with supplementary detail to the selected quotations provided in Table 2.

**Table 2:** Additional participant quotes as evidence of themes

Effects of medication	… it stops me from going like real sad and like just to those dark places, like real dark … (P5) I just start to get really, really, upset, and I just want to cry … I’m like damn it, I should have taken my pill today (P9) … I was taking it and it tasted disgusting in the back of my mouth and I wanted to vomit afterwards, but like that was it really. (P10) … it’s really hard to differentiate what’s the result of the medication and what’s the result of life circumstances in terms of my mood and anxiety (P11) … it’s [Fluvoxamine; antidepressant] stagnated a bit, and I’ve gotten to the point where I’m improving in some fields of my life, but there are parts where um things aren’t getting better or things are getting a bit worse (P17)
Finding the right medication	… So I went to a different GP … she said that I was on the lowest dose of Lexapro® [Escitalopram; antidepressant], I was only on 10 mg which wouldn’t help a mild anxiety let alone my issues … (P11) I’ve done some research into it and Paroxetine [antidepressant] is one of the worst ones to come off. Apparently, people have reported um discontinuation syndrome for 18 months afterwards and I was like, oh, that’s a fun fact [laugh] (P11) I’ve been on so many of them that it’s like I sometimes have to think like you know just how many of them are actually available to how many I’ve actually been on (P13) Um, the sheer anxiety of changing over to a new antidepressant, honestly like has - I would have done this much earlier if I didn’t have to think about it, because in the past I’ve had a doctor tell me just to go cold turkey … I honestly could not take the symptoms, so I just gave up and took it that day. Like I think I lasted like 3 h (P14)
The cost of being well	It’s annoying. Um, I wish I didn’t have to cause it’s just like I have to constantly remind myself to take it and if I don’t take it then, you know, I get all unbalanced and, my emotions are all over the place … (P9) … I’m on sort of four different medications and it gets like you know - I’m like 24, like I feel like I’m 64 … I know people who aren’t taking any medication and it would just be nice not to have to remember all that stuff (P14) … I believe in free healthcare. I think it should be through taxes and I don’t think that it should be something that people can’t afford. You know health is not something you should profit from (P17)
Routine medication use	… cause mum is really like you know wants me to get better and everything, so every night she’d give me my Zoloft® [sertraline], and I’d have to lift up my tongue and show her that I’d taken it so … I think I was like brought up kind of, you know, you need to take your medication it’s not like a one, two, skip a few type thing (P2) … it’s also really hard for me take the medicine because I’m so forgetful, but, I also have my dad reminding me like every morning, he’s like oh have you taken your tablet and I’m like no and he’s like get in there (laughs) (P5) … I was being stubborn, I was like I don’t need them I can just do well on my own but, I can’t (P4)
Being believed and supported	… I kind of just feel like I’m going to be judged because I’m a young person needing to taking it [medication] for my mental health (P3) … [Mother] had a big go at me because she thought that they just mess with your head even more, and you have to rely on them all the time, and they don’t actually work or something … I stopped using them … (P5) I don’t know I guess I just thought that after a little while I’d just start to go away and I wouldn’t need it, you know like when you get sick and you get antibiotics and you’re only on it for so long and then your problems go away … (P9) I have a few people that have told me, no, don’t take it. Don’t go on it, and everything, but I kind of tune it out at the same time, because I feel like it is doing me like really well and stuff being on medication (P15) I think when you’re so depressed that you feel like you could die any moment, you don’t have the luxury of being able to be like, what if? You know, it’s kind of like, this is my option and I’ve just got to do it (P17)

## Results

Eighteen participants between the ages of 14 to 25 were interviewed; the majority were female (*n* = 16) and over the age of 16, with two participants aged 15 and 14 (Table 3). All but three participants were living with depression or anxiety as their primary concern; one participant did not specify their condition and two participants had been diagnosed with bipolar disorder. Eight participants described experiencing both anxiety and depression, two of which had an associated eating disorder. The length of time participants had been using their current medication varied from 3 months to 3 years. Eleven participants disclosed specific details of medication use; antidepressants were most commonly described across the entire study sample. All 18 interview transcripts were included in the data analysis.

**Table 3:** Additional participant demographics^a^

Code	Gender	Mental Illness/es referred to	Duration of medication use
*P1*	F	anxiety, depression, OCD	approx. 2.5 years
*P2*	F	anxiety, depression, bipolar disorder, eating disorder	variable use over approx. 9 years
*P3*	F	anxiety, depression	11 months
*P4*	F	not specified^b^	6 months
*P5*	F	anxiety, depression	12 months
*P6*	F	depression	not reported
*P7*	F	anxiety	approx. 3 months
*P8*	M	anxiety, depression	current medication 4 months, trialled other medication previously
*P9*	F	not specified^c^	nearly 3 years
*P10*	F	depression	1.5 years
*P11*	F	anxiety, depression	current medication 7–8 months, trialled other medication previously
*P12*	F	anxiety, depression	variable use over 2 years
*P13*	M	bipolar disorder	not reported but mentioned using one medication for at least 12 months
*P14*	F	depression	approx. 6 years
*P15*	F	anxiety, depression	2–3 years
*P16*	F	Anxiety, depression	not reported
*P17*	F	PTSD/anxiety, depression	11 months
*P18*	F	Anxiety, depression, eating disorder	approx. 2 years

^a^ Details were deciphered from interview data only and must not be taken as complete information

^b^ Participant thought they did not have depression but bipolar disorder

^c^ Participant referred to a medicine that was used when needed for a panic attack/anxiety

Six key themes were identified in the data, and aspects of the Medication Experience Model were strongly supported. This included medication benefit (finding the right medication and effects of medication), side effects (effects of medication), burden (cost of being well), and medication adherence (routine medication use).

### Effects of medication

Medication use alleviated symptom severity and improved overall quality of life. Some participants reported that their mental health status had improved to the extent that their self-harming episodes had reduced. Participants generally agreed that medication helped them to function, although few imagined that it guaranteed complete improvement:

“You know it [medication] doesn’t cure everything … but it sort of helps keep me floating, you know, while I manage my problems” (P16)

“The lithium [mood stabiliser for bipolar disorder] - it’s shaved off the edges if that makes sense. I’m not as sharp in the moods. I’m not as high and not as low …” (P13)

It was noted that medication benefits could stagnate and become less useful over time; if symptoms did not completely resolve, some participants tended to question whether the right medication had been prescribed:

“I still don’t know if Pristiq’s® [Desvenlafaxine; antidepressant] the right one for me … it’s what actually did take me out of that darkness and bring me back to just functioning enough for every day, but … I still feel like there’s sort of just that barrier there that’s like stopping me from living my life” (P14)

Medication benefits were often offset by unwanted, sometimes distressing, side-effects, such as vivid dreams:

“… they [vivid dreams] can be quite frightening because I feel like I’m really there when I’m in the dream, and a lot of the time I feel like I’m half way between asleep and awake, but I can’t wake myself up” (P14)

Another participant referred to medication as a “*double-edged sword,*” explaining that whilst medication had helped to stabilise and normalise her life, she had concerns about her physical wellbeing:

“… it [medication] helps your mental health but at the same time it’s kind of detrimental because it’s making you fat, and making you sleepy, so you don’t want to exercise, you don’t wanna do any of that …” (P2)

Participants described a continuum of medication side-effects from initial, to established, use. While side-effects such as reduced concentration, weight gain and tiredness were generally unwanted and caused concern, a degree of sedation was nonetheless accepted by one participant because of improved sleep. Weight gain was a key issue for young people using antipsychotics; conversely, some medication could possibly work as an appetite suppressant which was problematic for one participant with history of an eating disorder. For participants with more than one mental illness, medication could impact both positively and negatively, such as improving depression but worsening anxiety symptoms. Feeling unwell due to medication non-adherence, such as forgetting a dose, was a frequently reported occurrence:

“… when I forget [antidepressants] that’s when I notice the change, the really anxious, like kind of almost paranoid type feeling, yeah …” (P12)

Participants used multiple ways to manage side-effects whilst continuing their medication, such as using anti-nausea agents or changing the timing of their medication dose. Self-talk was another such strategy with one participant stating:

“… just reminding myself that I’d rather be you know, a little bit chubbier and be happy than, you know, small and skinny and mentally unwell” (P16)

Treatment was life-saving for one participant; even though they had experienced significant side-effects from medication use, there were currently no viable alternatives. When asked if medication use had affected their personality, one participant disagreed, believing that this was more likely due to personal growth and development rather than from using her psychotropic medication. Other participants, however, raised concerns about the negative effect of medication on their personality, and becoming *“lifeless, emotionless*” (P13).

Whilst medication use was not ideal for most participants, many viewed it as better than being mentally unwell. Ultimately, participants sought medication changes when side-effects became burdensome, when the medication was considered ineffective, or when symptoms were exacerbated. The complex process of finding the right medication was a key focal point in the interviews.

### Finding the right medication

The decision to change medication was often fraught with anxiety; switching medication was widely considered a challenging and frustrating process of trial and error. However, for some young people, trialling a new medication was associated with optimism and hopefulness that they would find one that was effective:

“… it does feel really disheartening [trialling multiple medications], but I guess like there’s always that little glimmer of hope at the end of the tunnel, like, that something will work” (P6)

“… You start up on the next one and you kinda don’t know if it’s gonna work or not work or be any better, if it’s gonna do anything … it’s very annoying in terms of I haven’t, haven’t found the right one …” (P1)

Participants confirmed that they often trialled multiple medications, particularly when a diagnosis was yet to be established. It was evident from participant accounts that finding the right medication was both an inexact science and often a protracted process. This was especially the case if the GP was perceived to have limited expertise in the area of mental health:

“… I’ve had a lot of doctors in the past where they don’t know a lot about medication for depression at all, they’ll just sort of look at a textbook or a list on their computer and just say, this one looks good” (P14)

“My GP isn’t kind of a mental health person so it’s just kinda like we’ll try this one, and then if that doesn’t work we’ll try this one, if that doesn’t work we’ll try this one. So, it’s been a lot of just kind of trial and error with what I’m doing …” (P1)

A small number of participants recalled being told by a healthcare professional not to abruptly cease their medication. However, one participant described a situation in which she was advised by her GP to immediately stop her antidepressant,^2^ which resulted in severe withdrawal effects requiring hospitalisation:

“I vomited every day for a month or two … uncontrollable vomiting and just extreme suicidal ideation, so I was really upset with that … I cried in his [GP’s] office, telling him I felt suicidal and he took me off medication cold turkey, without warning me that it could make it worse. So yeah, I find that to be the worst experience I’ve had …” (P17)

There was the potential that a person’s mental illness could be exacerbated during medication change-over processes. Two participants (P1 and P11) took a proactive approach to this event and informed family and friends so that they were prepared for subsequent mood changes and behaviour. Participants generally recognised that there were no guarantees a new medication would be more effective than the current treatment, and unwanted effects could still be experienced as medication dosages were gradually reduced. Side-effects from medication changes were not the only concerns raised; financial burden specific to medication cost was an everyday reality for many participants.

### The cost of being well

The impact of medication cost was acutely felt by many participants, particularly if their prescribed medication was not subsidised by the Australian Government on the PBS.^3^ Medication costs were frequently viewed as exorbitant by a population with limited financial capital; participants were often studying and paying associated tuition and travel fees, living out of home and paying rent, too unwell to work, or a combination of these. Parents sometimes assisted with medication costs and participants recognised, and appreciated, this support. However, one participant felt guilty asking for financial support from a parent, resulting in delayed medication use and worsening symptoms:

“… if you don’t take it for a few days you, you go real low [be] cause, a while ago I ran out but I didn’t tell Dad because my medicines are really expensive” (P5)

Ultimately, being prescribed costly and unaffordable medication left participants with stark choices:

“… not trying me on a medication that’s on the PBS … and knowing full well that I’m struggling to pay rent … What am I supposed to do? Be homeless but buy my medication?” (P17)

Paying full-price to start a new medication was generally viewed as a waste of money, especially if the medication was discontinued after a brief period, for example due to unacceptable side-effects. Medication sharing with other household members was used to circumvent such expense when someone else had trialled the same medication and had some remaining:

“Or if you’re going on a trial - that’s something else because there’s been a lot of shifts in people’s stuff. It’s like well you may as well have half a packet of it in case it’s shit and you don’t want to buy it first up …” (P18)

This practice avoided medication wastage as the same medication had been prescribed, thus allowing the effect of the new medication to be assessed without the associated costs.

### Routine medication use

Most young people experienced little difficulty incorporating their medication into their daily lifestyle, with a variety of strategies used to help them to remember to take their prescribed dose at the right time. Prompts included: setting mobile phone alarms; keeping medication in the same, frequently used, place; and in multiple locations (partner’s and parental homes); using a self-prepared medication box or dose administration aid; writing important details on the back of the pill wrapper; and family members/friends providing daily reminders. Taking medication for a mental illness appeared to be easier for participants managing other chronic conditions, such as endometriosis, or using other medication. For example, one participant integrated taking her antidepressant medication with her oral contraceptive pill.

Participants described periodically forgetting to take their medication, especially if they had a tendency to be absent-minded or when using medication on an ongoing basis was a new experience. One participant disclosed that she stopped using her antidepressant because she wanted to experiment with recreational substances:

“… he [boyfriend] was like, you shouldn’t take antidepressants or SSRIs [Selective Serotonin Receptor Uptake Inhibitors; antidepressants] with them and so I was like, okay, I’ll just stop taking it … I hated acid [recreational substance] because it would made me feel so anxious and I’m never going to take that again, and probably none of the other ones again because I’m not interested … I was better off just being stable, happy, like on antidepressants” (P12)

Another participant confirmed that young people commonly seek alternative methods to escape their mental illness, including the use of recreational substances. Given that adolescence/early adulthood is associated with experimentation and risk-taking behaviour, credible information on medication interactions was viewed as an important contribution to minimising risk:

“… something that probably should be discussed, especially with youth, is like all of the different medication interactions with illicit drugs because the reality is like, the reality is people are doing drugs. Like there’s just not really a question about it …” (P18)

The importance of information to mitigate medication dilemmas, such as what to do in the event of missed doses, unwanted side-effects and accidental overdosing, was also discussed.

### Information needs

Participants generally considered themselves as accountable for their own wellbeing and most made their own decisions with respect to accepting, or rejecting, medication use for their mental illness. Access to quality information was integral to this:

“I feel like I need as much information as I can because I’m sort of the one, I’m the only one who is making my decisions for myself so I really want them to be informed” (P14)

Being well informed was repeatedly demonstrated by the many participants who referred to their medication and/or mental illness using the correct nomenclature during their interview. Information was identified as a key component in facilitating treatment autonomy, which could address misconceptions:

“… like it makes total sense people are so scared to do [i.e. take] medication because they don’t want to be zombies and they don’t want to be suicidal, and so it’s like, yeah, people don’t understand it. Don’t understand what it does, don’t understand the risks. Don’t understand what to do if the risks happen to you, either.” (P17)

While friends, family and Google© were all reported as valued sources of information, this did not replace the importance of obtaining in-depth advice from healthcare professionals. For one participant with bipolar disorder, a lack of information resulted in symptoms of lithium toxicity^4^ 2 months into her treatment. Additionally, no-one had advised her of the potential health risk of taking over-the-counter analgesics, such as non-steroidal anti-inflammatories, with her lithium:

“Well I was really shocked because I, no one had told me [about the drug interaction and associated risk], my psychiatrist didn’t tell me, my doctor that put me on it [lithium] initially didn’t tell me … I’d buy like Nurofen Plus® [Ibuprofen and Codeine; analgesics] like if I had really bad period pain, they’d [pharmacist] be like ‘Oh yeah, that’s fine’. Like they had dispensed my lithium, they knew I was on lithium, and no one had told me …” (P2)

For participants who had never previously used any medication on a regular basis, commencing medication for the first time for a mental illness was a particularly daunting experience. Reviewing lists of possible side-effects often resulted in hesitancy about medication use:

“… the side-effects kind of freaked me out at first … I really wasn’t sure because I’d never really taken that much medicine in the past … so yeah, it was kind of scary at first” (P11)

In addition to service providers failing to supply high quality information, other frustrations were raised by participants, including not being treated as a credible witness to their own wellbeing, feeling stigmatised, and the inconsiderate actions of other young people around them.

### Being believed and supported

Not all young people had the support they needed when considering treatment for their mental illness or had their situation taken seriously by older people, including healthcare professionals. For example, one participant recalled a situation when his psychologist did not believe that his feelings of exhaustion were related to sertraline [antidepressant] use:

“… he [psychologist] pretty much said this isn’t listed as a side-effect and therefore you can’t be experiencing that …” (P8)

Another participant stated that her mother disbelieved her mental illness, deciding instead that she was just going through a typical ‘teenage stage’ and questioned the need for medication. The family unit often influenced whether, and to what degree, young people accepted a mental illness diagnosis and medication use. Family units with members who had a positive lived experience of mental illness tended to be more accepting of the young person’s need for medication. Incorrect parental assumptions, or beliefs, about mental illness or the necessity of medication, often delayed help-seeking or resulted in premature medication cessation:

“Yeah I think I’m one of the lucky ones, but I know a lot of people, a lot of my friends who have similar issues and would never take medication because their parents think it’s stupid or their parents don’t think it works or, they think that yeah you just need to suck it up and move on and, it’s all in your head …” (P7)

Telling loved ones about their mental illness or medication use was not an easy task, even for participants with loving and understanding families. Some participants sought support outside the family unit; living with other young people with a mental illness was reported as normalising and mutually beneficial. However, while a supportive household could be helpful, participants also urged caution to avoid becoming over-involved with other young people:

“Like if your friend is going through something really, really, hard obviously be there for them. But … if that’s affecting your healing and your journey with medication and with mental health sometimes you just need to shut the door. Like, politely shut it …” (P16)

This participant also raised concerns about the increasing rates of self-diagnosis and the attention seeking behaviours evident from social media posts which trivialised those young people with a diagnosed mental illness. Another participant confirmed:

“… when people say they have depression like you help them as much as you can because you know how it feels, but if they don’t actually have it and they just want attention like she [fellow school student] did, it just messes with your head” (P5)

While there was evidence of stigma from participant conversations, particularly in relation to the work environment, there was a sense that that this was reducing. Regardless, most participants did not actively disclose their condition and/or hid medication use from others. One participant described feeling grateful that they did not have to take their medication at school and having to justify being called to the office every day to obtain their medication. Another participant explained that they did not want to disclose their situation, not from a sense of shame, but to avoid being bombarded with questions. Others raised concerns that people would perceive them as weaker or that they would be viewed differently if their mental illness was made public knowledge.

## Discussion

Young people persevered with finding the right medication for their mental illness, within a complex and challenging healthcare system that tends to position these consumers as more vulnerable, not least because their living environments tend to be more fluid (i.e. less stable). Our study participants appeared highly independent and self-motivated individuals; to a certain extent this was required when support from healthcare professionals and the overall health system was missing. Medication use was particularly difficult if the GP had limited expertise in this therapeutic area or viewed the young person as a passive, rather than an active, participant in their own welfare. This study also provides further insight into the costs associated with being well, specifically relating to medication costs for a population that is already at a financial disadvantage. Other societal challenges were identified, such as managing one’s mental illness within the (social media) context of other young people who self-diagnose their condition or seem less unwell. Additionally, several challenges previously reported in the literature were recognised, such as the tenuous balance between medication benefits and side-effects [30, 42], concerns related to disclosing medication use and/or the associated diagnosis [21, 43], and medication trial and error [15, 16]. Young people with mental illness are juggling several issues which need to be recognised and better supported by healthcare policy and service delivery.

### Policy implications

This study identified that financial burden related to medication costs, particularly for medications not subsidised by the Australian Government, was a significant concern. In some instances, this placed young people in a tenuous position, particularly if they did not have the financial support from other family members. Some participants undertook practical solutions to manage this, such as strategically missing medication doses, or stockpiling and sharing medication with housemates. Conversely, relying on parental support to pay for medication use could inhibit a young person’s sense of autonomy and self-management. Although financial burden was recognised by Werremeyer et al. [39], this has not been widely investigated and reported in this population. Yet, financial burden has been identified as a barrier to medication adherence in older people using antidepressants [44]. Our study findings suggest that Australia’s medicine policy could be strengthened to better support young people, as evidenced by the actions taken by other countries. For example, financial reimbursement for medication used to manage severe and persistent mental illness, such as psychoses, is provided for children and adolescents in Finland [45]. In countries with similar healthcare systems to Australia, medications are free in Scotland, Wales and Northern Ireland and for young people under 16 years in England [46]. There is no prescription co-payment for children under 13 years in New Zealand; all other subsidised medications cost five dollars (NZ) for up to 3 months’ supply [47]. Australia’s medication costs are significantly more expensive, which has not changed since being stipulated as a ‘bad deal’ by the Grattan Institute in 2013 [48]. Unless Australian medication policy changes, healthcare professionals have a responsibility to consider this issue, and inform recipients, when prescribing, or supplying, medication that has significant out-of-pocket costs [49].

### Practice implications

The conduct of some healthcare professionals can intensify the vulnerability of young people using psychotropic medication. In addition to prescribing unaffordable medication, there were other examples of inadvertent harm arising from a lack of information or inappropriate directions being provided, which has been recognised elsewhere [42]. Lack of care was evident in the case of one participant who experienced significant withdrawal effects from following her GP’s medical advice to abruptly cease her prescribed medication. Such examples strengthen the call for healthcare professionals to actively participate in optimising opportunities for medication information provision [50], and to seriously consider the specific needs and particular concerns of this population [51].

The young people in our study were mostly experienced medication users, and subsequently, demonstrated high self-efficacy with respect to managing their medication and remembering to take doses as prescribed. They generally displayed high level self-management strategies, as exemplified by the actions of one young person who decided to stop their antidepressant to trial recreational substances. While such actions have been acknowledged as problematic in relation to antidepressant adherence [44], in a practical sense, by choosing not to use both drug types together, this participant demonstrated a somewhat responsible attitude and high level of self-efficacy. Our study findings also add to the scant body of literature on the resistance of self-stigma [52, 53]. This was shown by participants’ perseverance with medication use. Although there were certainly frustrations with having trialled multiple medicines, there was no sense of self-blame when medication did not work out as planned. Indeed, blame was sometimes attributed to the prescriber. This did not necessarily mean that young people did not experience or have stigma-related concerns, as demonstrated by participants who were less forthcoming about disclosing their medication use. This was particularly evident in relation to family members [54]. While being around others with lived experience of mental illness was mostly perceived as helpful, caution was advised regarding the potential for negative impact on an individual young person’s mental illness. Concerns were also articulated with respect to the actions of peers without a definitive mental illness diagnosis who used social media to gain empathy. While a lack of personal experience has been identified as a barrier to understanding and empathy [55], our study highlights that this needs to be a genuine experience.

Although this study was not focused on exploring treatment burden, there was evidence of this issue with respect to medication burden, such as costs and side-effects, and healthcare access burden, for example, unhelpful relationships with healthcare professionals [23]. The concept of treatment burden is gaining increased attention with a recent development of a conceptual framework to measure impact in complex patients with chronic conditions [56], and the Patient Experience with Treatment and Self-Management (PETS) tool to measure self-reported treatment burden [57]. While patient-reported outcome measures (PROMs) are increasingly recognised as a way to assess healthcare quality, they are not currently mandated in Australian healthcare policy [58]. Furthermore, the work involved in creating PROMs, such as the PETS tool, have focused on older people and not young people. Further research is needed to validate the use of such measures and/or other work on treatment burden for this specific population. The findings of the study protocol by Barbic and colleagues in estimating the extent to which available PROMs are fit to measure the mental health and recovery needs of youth, will be particularly useful [59]. In the meantime, our study findings advocate for healthcare professionals to consider how they might try to understand the lived experiences of the young people who come to them for mental health support.

### Research implications

This work also provides findings that align with the ‘medication experience’ domain of the Medication Experience Model described by Werremeyer and colleagues [39]. Our study participants described experiences related to medication benefits, side-effects, burden, adherence and a lack of information. This is valuable information given that the model was originally based on the findings of participants with a median age of 35 years [39]. However, there were some nuances, which may have been a result of the different methodology used, the interview questions and broader study aim. For example, medication side effects and benefits, which were separate themes in the model, were inexplicably linked, with some of our study participants experiencing both effects simultaneously. Frustration with the trial and error approach was a key issue reported by our participants, particularly when changes could cause side effects such as withdrawal or exacerbation of one’s mental illness. Subsequently, this was not coded as a medication benefit as seen by Werremeyer and colleagues [39]. Finally, our data showed that there were instances of being disbelieved by others, which was not reported in the model [39]. Therefore, further research is needed to validate the Medication Experience Model in this population, particularly with young people from broader cultural and/or ethnic backgrounds and age groups. Whether, and how, this model relates to, or integrates with, the overall concept of treatment burden also requires further exploration.

### Study limitations

There are limitations to this exploratory study. To optimise the voluntariness of participation, young people were not required to disclose details about their mental illness diagnosis nor their treatment regimens; we agree that this information would have provided additional insight about the study sample. While this is a key study limitation, the research team were cognisant of the vulnerabilities of the population they were interviewing and wished to do what they reasonably could to maximise their anonymity. As only single, one-off, interviews were conducted, opportunities for the researchers to build rapport with participants were limited [60] and therefore, not all relevant information may have been obtained. Participants, while representing a variety of life experiences, were mostly female and Caucasian (although this information was not confirmed by asking participants to provide broader demographic details such as sexual orientation, ethnicity etc.) and thus findings may not be generalisable to young men and those from culturally and linguistically diverse backgrounds. The sample was relatively homogenous with respect to the mental illnesses represented, which, when reported, were predominantly depression and anxiety. Interviews with young people experiencing more severe or persistent mental illness is warranted, as well as those in the early years of adolescence. Lastly, the majority of participants were not newly diagnosed with a mental illness and therefore concerns related to new medication use was not provided. The researchers were unable to identify more nuanced themes between participants in relation to their level of medication experience with mental health medications or more generally.

## Conclusion

Mental illness remains a key health priority area in Australia, particularly for young people managing the challenges of taking psychotropic medication. Young people persevered with taking medication to manage a mental illness within a healthcare system that does not adequately support this vulnerable population with limited financial capacity. Further to this, there remains a clear directive for healthcare professionals to provide credible information to young people and to engage with them as active participants in their own healthcare. This includes treating young people as an active participant in the treatment process, being aware of the financial burden and capacity for young people to pay for medication, optimising opportunities for the provision of medication information, and attention to therapeutic decision-making in relation to prescribing.

## Data Availability

The research data is stored securely as per ethics approval at Griffith University and cannot be made publicly available. The authors will consider any reasonable request for access to the anonymised data according to the privacy statement provided with participant information and consent materials. Please direct requests to the corresponding author.

## Abbreviations

PBS: Pharmaceutical Benefits Scheme
PETS: Patient Experience with Treatment and Self-Management
PROMs: Patient-Reported Outcome Measures
GP: General Practitioner

## Footnotes

1 The Australian Government subsides the medications listed on the Pharmaceutical Benefits Scheme (PBS); some of these medicines are priced below the co-payment and therefore paid entirely by the consumer.

2 Immediately stopping psychotropic medication is often referred to as ‘cold turkey’ and can result in withdrawal side-effects such as electric-shock like sensations, anxiety and sleep disturbances. A gradual withdrawal process is recommended with variable length of changeover before initiating a new medication (depending on the medication class). Source: Australian Medicines Handbook (AMH), 2019, Australian Medicines Handbook Pty Ltd.

3 Not all psychotropic medications are listed on the PBS, meaning that consumers are required to pay a non-standardised price, which can vary between community pharmacies.

4 Lithium, a mood stabiliser, is a narrow therapeutic index medication; there is a small margin between beneficial and toxic effects. Some medicines, such as non-steroidal anti-inflammatories can increase the levels of lithium in the body placing a person at increased risk of lithium toxicity. This results in symptoms such as vomiting, diarrhoea and profuse sweating.
